# Overexpressed DEPDC1B contributes to the progression of hepatocellular carcinoma by CDK1

**DOI:** 10.18632/aging.203016

**Published:** 2021-05-25

**Authors:** Xiao-Wei Dang, Qi Pan, Zhen-Hai Lin, Hao-Hao Wang, Lu-Hao Li, Lin Li, Dong-Qi Shen, Pei-Ju Wang

**Affiliations:** 1 Department of Hepatopancreatobiliary Surgery, The First Affiliated Hospital of Zhengzhou University, Zhengzhou, Henan, China; 2 Department of Hepatic Surgery, The Cancer Hospital of Fudan University, Shanghai, China

**Keywords:** DEPDC1B, CDK1, proliferation, migration, hepatocellular carcinoma (HCC)

## Abstract

Background: Hepatocellular carcinoma (HCC) is the main type of primary liver cancer and shows a heavy burden worldwide. Its recurrence and mortality rate are still uncontrolled by the usage of present treatments. More attention has been focused on exploring specific genes that play important roles in HCC procession, and the function of DEP domain containing 1B (DEPDC1B) in HCC has not been researched.

Methods: Immunohistochemical staining was used to detect the expression level of DEPDC1B in tumor tissues and adjacent normal tissues. After DEPDC1B and CDK1 knockdown in cell lines HEP3B2.1-7 and SK-HEP-1, MTT assay and colony formation assay was used to detect cell growth, flow cytometry assay was used to investigate cell apoptosis and cell cycle, wound-healing assay and Transwell assay were used to examine the tumor cell migration. Moreover, a xenograft model was constructed to research functions of DEPDC1B in tumor growth *in vivo*.

Results: The results show that DEPDC1B knockdown inhibit the progression of HCC, through inhibiting cell proliferation, migration, colony formation, leading to G2 phase arrest, and promoting cell apoptosis *in vitro,* and CDK1 was selected for further mechanic research according to the results of Human GeneChip prime view. The results of recovery experiment displayed that the functions of DEPDC1B on HCC progression were mediated by CDK1. DEPDC1B knockdown can also inhibit tumor growth *in vivo*.

Conclusions: The study confirmed that DEPDC1B knockdown restrains the tumor growth *in vitro* and *vivo*, and it can interact with CDK1 and rescued by CDK1. The study suggested that DEPDC1B was as a potential therapeutic target involved in HCC growth and progression.

## INTRODUCTION

Liver cancer is the fourth biggest cause of mortality around the world in 2018, and the sixth most commonly diagnosed cancer, about 841000 new cases and 782000 deaths each year [1]. Hepatocellular carcinoma (HCC) is the main type of primary liver cancer, accounts for 75%-85%, which shows a heavy burden worldwide. The main cause of HCC consists of chronic hepatitis B virus (HBV) infection, hepatitis C virus (HCV) infection, aflatoxin, aristolochic acid, alcohol abuse, and metabolic syndrome related to obesity and diabetes, and the major risk factors are various based on the region [1–5]. Although various treatments for HCC have been used to help patients, the severe criteria and high recurrence for resection, and low sensitivity of chemotherapy do not satisfy well the requirement of extending the life expectancy of them. Even if the surgical resection which is recommended in early-stage patients, the five year survival is 50%, the recurrence rate accounts for 70% [6]. For the purpose of overcoming the dilemma, more attention has been focused on the mechanism of HCC to find specific genes that play important roles in HCC procession [3, 7, 8].

DEPDC1B (Homo sapiens DEP domain containing 1B) is a human protein which is encoded by *DEPDC1B* gene located on chromosome 5 (5q12.1), and widely expressed throughout human tissue. However, the functions of DEPDC1B have not been investigated precisely. Study showed that DEPDC1B can accumulate in G2 phase to cooperate with de-adhesion events and cell-cycle progression at mitotic entry by inhibiting RhoA signaling, and it also can regulate cell proliferation in zebrafish embryogenesis [9]. Although the precise function of DEPDC1B is uncertain, it is reported that DEPDC1B knockdown can inhibit tumor growth in malignant melanoma and some other types of malignant tumors [10, 11]. However, the functions of DEPDC1B in HCC have not been investigated. Therefore, the study aimed to discover the functions and general mechanism of DEPDC1B in HCC to instruct the therapy of HCC. If the functions of DEPEDC1B is studied in depth, the treatment methods and effects of HCC will be expanded and enhanced. To some degree, through further research, the study will solve the current problems in the treatment of HCC.

## RESULTS

### DEPDC1B was overexpressed in tumor tissues and related to advanced stage

**Figure 1:**
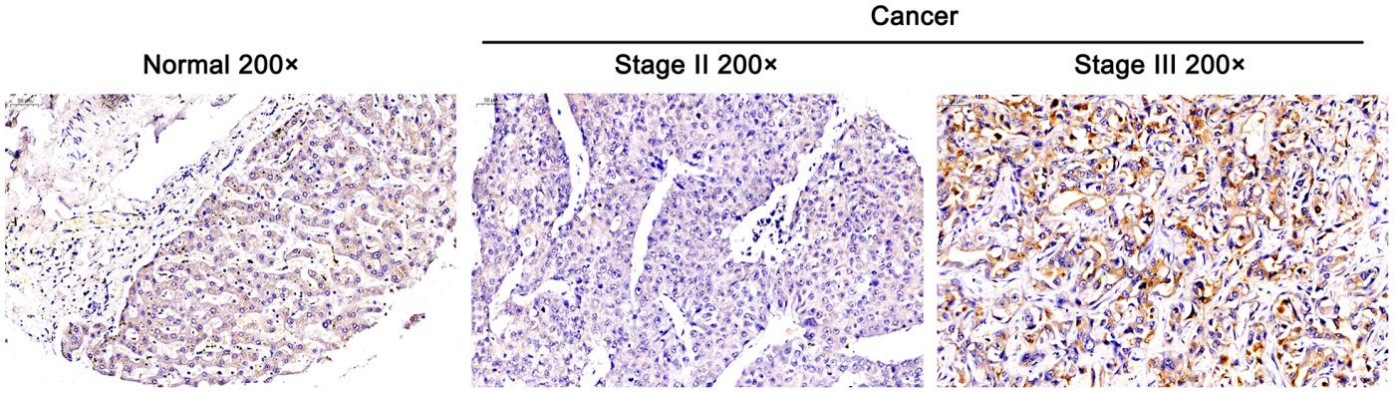
**Overexpressed DEPDC1B in tumor tissues and it was related to advanced stage.** The expression of DEPDC1B was detected by immunohistochemistry and overexpressed in cancer tissues compared with normal tissues (Magnification ×200, scale bar = 50 μm).

The general information, tumor stage and T stage of these cases were collected in Table 1. The results of immunohistochemistry revealed that DEPDC1B was overexpressed in HCC tissues compared with para-carcinoma tissues (Figure 1 and Table 2), and the expression level of DEPDC1B was significantly associated to the pathological stage of tumors and T stage (P <0. 05) (Table 3), suggesting that DEPDC1B was overexpressed in tumor tissues and related to advanced tumor stage. The results of TCGA about lung adenocarcinoma showed that the expression level of DEPDC1B was significantly correlated with the pathologic T, pathologic N, tumor stage, and gender (Table 4).

**Table 1:** Relationship between DEPDC1B expression and tumor characteristics in patients with hepatocellular carcinoma.

**Features**	**No. of cases**	**DEPDC1B expression**		***P* value**
		**low**	**high**	
All cases	178	104	74	
Age (years)				0.117
≤50	91	48	43	
>50	87	56	31	
Gender				0.662
Male	144	83	61	
Female	34	21	13	
Grade				0.460
1	21	8	13	
2	125	79	46	
3	22	11	11	
Stage				0.001
I	6	4	2	
II	78	56	22	
III	94	44	50	
T stage				0.003
T1	6	4	2	
T2	80	56	24	
T3	86	42	44	
T4	6	2	4	

**Table 2:** Expression patterns in hepatocellular carcinoma tissues and para-carcinoma tissues revealed in immunohistochemistry analysis.

**DEPDC1B expression**	**Tumor tissue**		**Para-carcinoma tissue**		***P* value**
	**Cases**	**Percentage**	**Cases**	**Percentage**	
Low	104	58.4%	20	100%	0.000
High	74	41.6%	-	-	

**Table 3:** Relationship between DEPDC1B expression and tumor characteristics in patients with hepatocellular carcinoma.

		**DEPDC1B**
Stage	Pearson Correlation	0.243
	Significance (Two-tailed)	0.001
	N	178
T stage	Pearson Correlation	0.224
	Significance (Two-tailed)	0.003
	N	178

**Table 4:** The relationship between the expression of DEPDC1B and tumor characteristics in patients with lung adenocarcinoma under TCGA.

		**DEPDC1B expression**		**No.**	***P***
		**Low**	**High**		
Pathologic_T	T1	100	67	167	0.010
	T2	112	147	259	
	T3	23	22	45	
	T4	10	9	19	
Pathologic_N	N0	172	146	318	0.017
	N1	38	53	91	
	N2+N3	28	44	72	
Pathologic_M	M0	156	168	324	0.119
	M1	8	17	25	
	M2	0	0	0	
Stage	Stage_I	149	114	263	0.008
	Stage_II	50	66	116	
	Stage_III	33	47	80	
	Stage_IV	9	17	26	
Gender	female	154	112	266	0.000
	male	93	134	227	
Age	low	122	128	250	0.557
	high	125	118	243	

### Establishment of DEPDC1B knockdown cell lines

**Figure 2:**
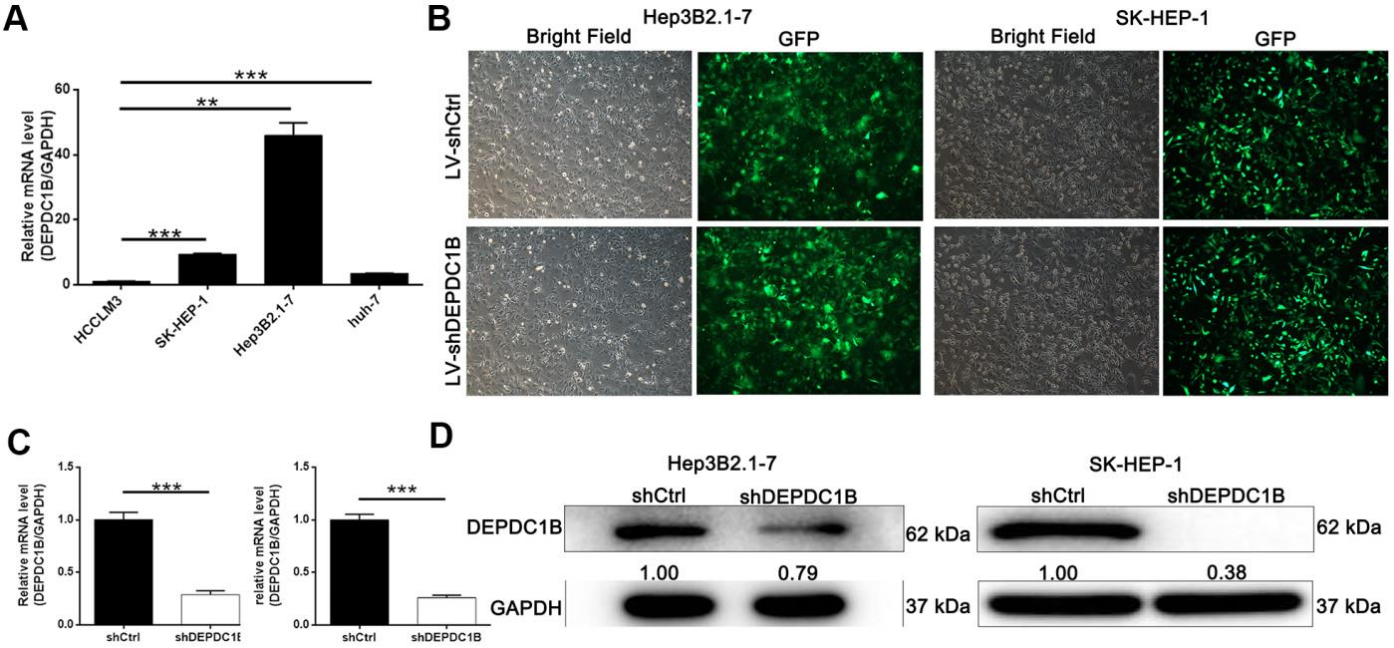
**Successful construction of DEPDC1B knockdown.** (**A**) The expression level of DEPDC1B in SK-HEP-1, Hep3B2.1-7, huh-7 was significantly higher than in HCCLM3 (P<0.05). (**B**) The fluorescence of cells, which were infected with shCtrl, shDEPDC1B for 72 h, observed by microscope demonstrates a >80% efficiency of infection and the normal cell condition. The results of qRT-PCR show that, after the infection of lentivirus: (**C**) Compared with the shCtrl group, in HEP3B2.1-7 cells, the knockdown efficiency of DEPDC1B in shDEPDC1B group was 71.35% (P<0.001) and in SK-HEP-1 cells, the knockdown efficiency of DEPDC1B in shDEPDC1B group was 74.1% (P<0.001). The results of Western blot show that, after the infection of lentivirus: (**D**) compared with shCtrl group, in HEP3B2.1-7 and SK-HEP-1 cells: the protein level of DEPDC1B in shDEPDC1B group was down-regulated. *: P <0.05. **: P <0.01. ***: P <0.001.

The results of qRT-PCR showed that the expression level of DEPDC1B in HEP3B2.1-7, SK-HEP-1 and huh-7 cell lines was significantly higher, compared to HCCLM3 cell line (*P*<0.05) (Figure 2A). The results indicated that HEP3B2.1-7 and SK-HEP-1 cell lines were more appropriate to research the functions of DEPDC1B. For the purpose of studying the effects of DEPDC1B knockdown, LV-shDEPDC1B and LV-shCtrl were transfected into HEP3B2.1-7 and SK-HEP-1 cell lines. After infected for 72 h, the efficiencies of infection in all group were more than 80% under fluorescence microscope in the way of detecting the fluorescence of cells (Figure 2B). The results of qRT-PCR displayed that the knockdown efficiencies of DEPDC1B of HEP3B2.1-7 and SK-HEP-1 cell lines in shDEPDC1B group were 71.35% (*P*<0.001) and 74.1% (*P*<0.001), compared with shCtrl group, respectively (Figure 2C). The expression level of DEPDC1B in shDEPDC1B group were down-regulated obviously compared with DEPDC1B in shCtrl group by western blot (Figure 2D). DEPDC1B knockdown cell models were constructed successfully according to the results of qRT-PCR and western blot.

### Knockdown of DEPDC1B inhibited HCC cell proliferation, migration and promoted HCC cell apoptosis and led to G2 phase arrest

**Figure 3:**
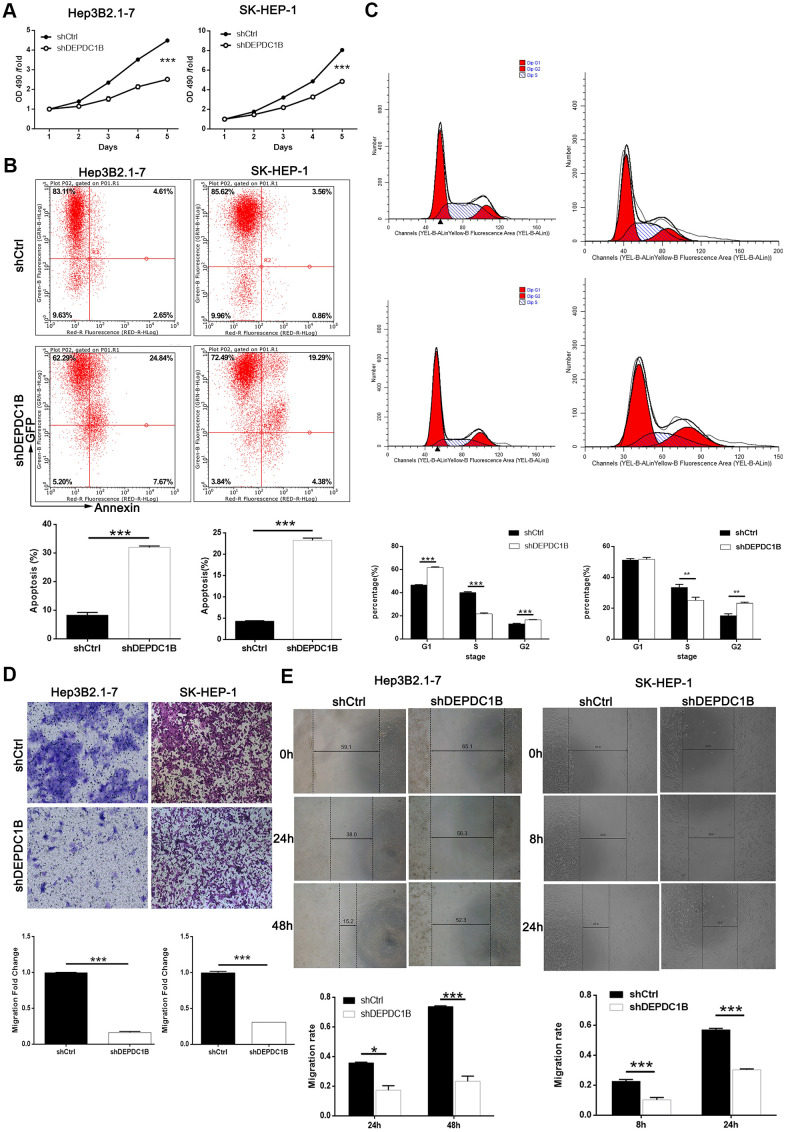
**DEPDC1B knockdown inhibited HCC cell progression *in vivo*.** (**A**) The results of MTT assay show that, after the infection of lentivirus: compared with shCtrl group, the cells in shDEPDC1B group exhibited slower proliferation rate (P<0.001). (**B**) The results of flow cytometry demonstrate that, after the infection of lentivirus: compared with shCtrl group, apoptosis percentage was increased in shDEPDC1B group (P<0.001). (**C**) The results of flow cytometry show that, compared with shCtrl group, in shDEPDC1B group, the percentage of SK-HEP-1 cells in G2 phase increased (P<0.01), and the percentage of Hep3B2.1-7cells in G2 phase increased significantly in shDEPDC1B group (P<0.001). Transwell assay showed that, after the infection of lentivirus: compared with shCtrl group, the migration ability of cells in shDEPDC1B group was inhibited (P<0.001) (**D**). (**E**) The results of wound-healing assay showed that, in HEP3B2.1-7 and SK-HEP-1cells, compared with shCtrl group, the migration rate of cells in shDEPDC1B group was decreased by 68% (P<0.001) and 47% (P<0.001). **: P <0.01. ***: P <0.001.

MTT assay was performed to study the effects of DEPDC1B knockdown on HCC cell proliferation, and the results showed that the proliferation in shDEPDC1B group was slower than that in shCtrl group (*P*<0.001) (Figure 3A). The results of flow cytometry assay exposed that the percentage of apoptotic cells in shDEPDC1B significantly increased compared with shCtrl (*P*<0.001) (Figure 3B). Further study in cell cycle showed the cells in G2 phase had significant increase in shDEPDC1B group compared with shCtrl group after cultured for 5 days (Figure 3C). Transwell assay and wound-healing assay were constructed to investigate the migration. Transwell assay showed that, compared to shCtrl group, the migration ability of HEP3B2.1-7 cells (48 h) and SK-HEP-1 cells (24 h) in shDEPDC1B were significantly decreased (*P*<0.001) (Figure 3D). The results of wound-healing assay showed that the average migration rate of HEP3B2.1-7 cells (48 h) and SK-HEP-1 cells (24 h) in shDEPDC1B group were decreased by 68% (*P*<0.001) and 47% (*P*<0.001), compared with shCtrl group (Figure 3E). Collectively, knockdown of DEPDC1B suppressed HCC progression by inhibiting cell proliferation and migration, promoting HCC cell apoptosis and leading to G2 phase arrest.

### Exploration of potential mechanisms

**Figure 4:**
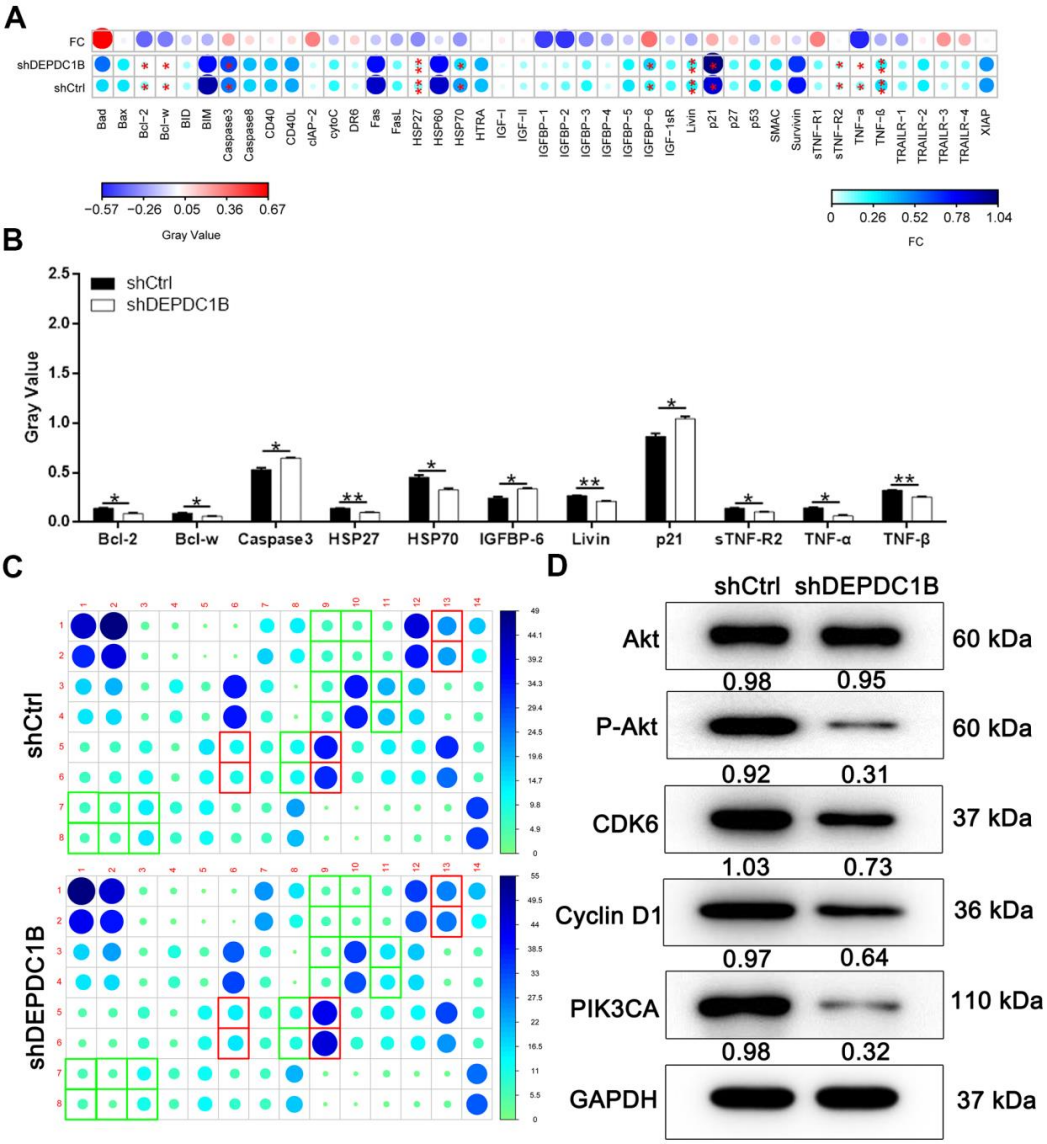
**Potential mechanisms of DEPDC1B functions on HCC.** (**A**–**C**) Bcl-2, Bcl-w, HSP27, HSP70, Livin, sTNF-R2, TNF-α, and TNF-β was significantly down-regulated in shDEPDC1B (P<0.05). (**D**) P-Akt, CDK6, Cyclin D1, and PIK3CA was down-regulated in shDEPDC1B group.

Human apoptosis antibody array was used to search for the affected proteins in the SK-HEP-1 cells infected with LV-shDEPDC1B and LV-shCtrl, because the specific function mechanism of DEPDC1B knockdown in cell growth was unclear. The expression of Caspase 3, p21, and IGFBP-6 was significantly up-regulated in shDEPDC1B compared with shCtrl (*P*<0. 05). The expression of Bcl-2, Bcl-w, HSP27, HSP70, Livin, sTNF-R2, TNF-α, and TNF-β was significantly down-regulated in shDEPDC1B, compared to shCtrl (*P* <0.05) (Figure 4A–4C). The results of western blot in SK-HEP-1 cells showed that the expression of P-Akt, CDK6, Cyclin D1, and PIK3CA was down-regulated in shDEPDC1B group, compared to shCtrl group (Figure 4D).

### DEGs were obtained by human GeneChip PrimeView and examined for further mechanisms

**Figure 5:**
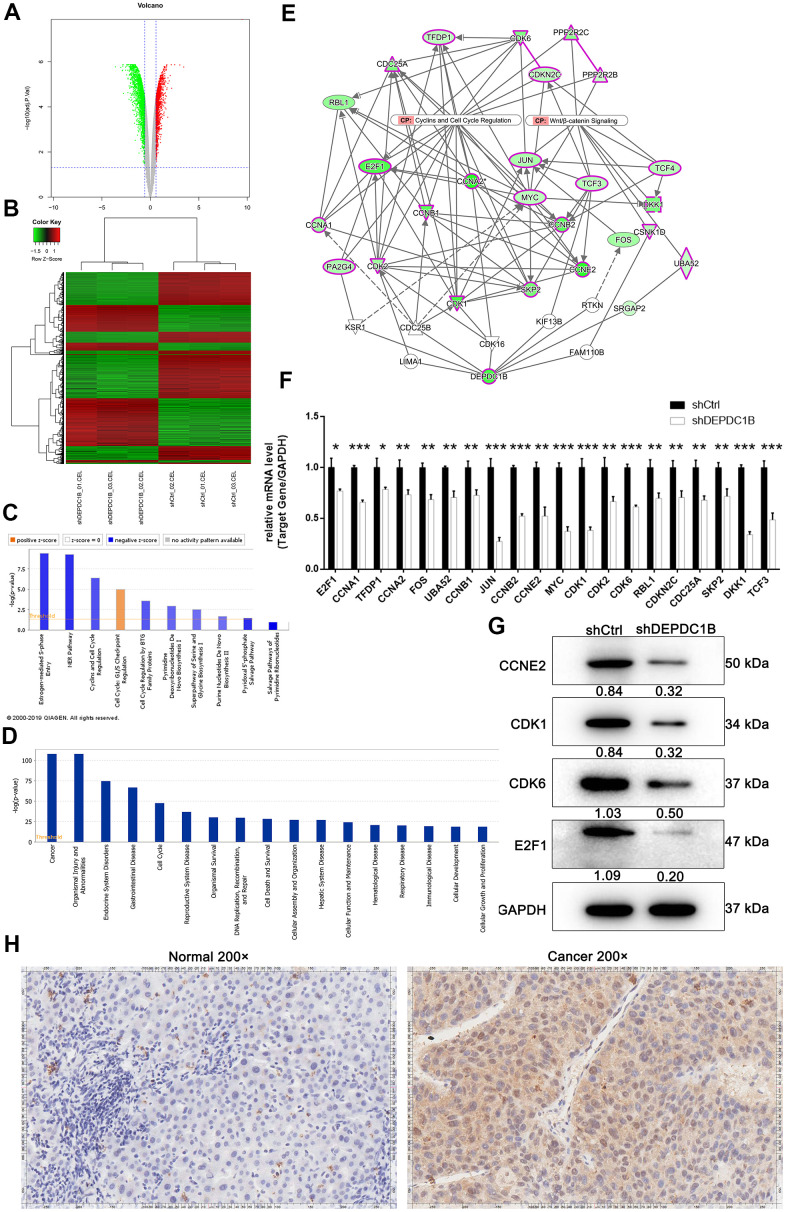
**Obtained the DEGs by human GeneChip PrimeView and the expression at mRNA and protein level.** (**A**) Genes were represented as red points or green points, when Fold Change ≤-1.5 and FDR<0.05 or Fold Change ≥1.5 and FDR<0.05. (**B**) Up-regulated genes and down-regulated genes were labeled as red and green, respectively. (**C**) Salvage Pathways of Pyrimidine Ribonucleotides, NER Pathway, Estrogen-mediated S-phase Entry, and Cyclins and Cell Cycle Regulation were significantly inhibited. (**D**) The enrichment of DEGS in disease and function. (**E**) The interaction network between DEPDC1B, Cyclins and Cell Cycle Regulation, and Wnt/β-catenin Signaling pathway. (**F**) The results of qRT-PCR showed that, compared to shCtrl group, the expression level of E2F1, CCNA1, TFDP1, CCNA2, FOS, UBA52, CCNB1, JUN, CCNB2, CCNE2, MYC, CDK1, CDK2, CDK6, RBL1, CDKN2C, CDC25A, SKP2, DKK1, and TCF3 was down-regulated (P<0.05). (**G**) The results of western blot showed that CCNE2, CDK1, CDK6, and E2F1was significantly down-regulated in shDEPDC1B group, compared to shCtrl group. (**H**) The results of IHC showed that the expression level of CDK1 in cancer tissues was higher than in normal tissues (scale bar = 50 μm).

For the purpose of exploring the molecular changes after DEPDC1B knockdown, transcriptomes between shCtrl cells and shDEPDC1B cells were compared by RNA-seq. The results of volcano plot and clustering analysis showed that 1063 DEGs (differentially expressed genes) were upregulated and 1320 DEGs were downregulated (Figure 5A, 5B). IPA pathway enrichment analysis of DEGs showed that DEGs were mostly enriched in Salvage Pathways of Pyrimidine Ribonucleotides, NER Pathway, Estrogen-mediated S-phase Entry, and Cyclins and Cell Cycle Regulation pathway and the three pathways were significantly inhibited, which indicated that DEGs played important suppressive roles in these pathways (Figure 5C). The IPA analysis about disease and function showed that cancer was one of the mostly enriched diseases (Figure 5D), which suggested that the DEGs found in the experiment are mostly relevant to cancers. DEPDC1B could affect CCNA1, JUN, CCNB2, CCNE2, CDK1, CDK6, etc in Cyclins and Cell Cycle Regulation, and Wnt/β-catenin Signaling pathway through EED and FANCD2 (Figure 5E). Further qRT-PCR showed that the expression levels of CCNA1, JUN, CCNB2, MYC, CDK1, and CDK6, were down-regulated in shDEPDC1B group, compared to shCtrl group (*P*<0.001) (Figure 5F). The results of western blot showed that the expression of CCNE2, CDK1, CDK6, and E2F1 was significantly down-regulated in shDEPDC1B group, compared to shCtrl group (Figure 5G). Collectively, the DEPDC1B knockdown suppressed the expression of CCNE2, CDK1, CDK6, and E2F1 at mRNA or protein level through Cyclins and Cell Cycle Regulation, and Wnt/β-catenin Signaling pathway. Among the several selected DEGs, CDK1 was found to be overexpressed in tumor tissues, compared with para-carcinoma tissues by IHC analysis (Figure 5H).

### Establishment of CDK1 knockdown and DEPDCB overexpression

As shown in the former article, CDK1 and CDK2 played important roles in perturbations of chromosomal stability, S phase and G2/M entry, which led to the tumorigenic events happen [12]. In order to further investigate the functions of CDK1 in cells lines and specific relationship between DEPDC1B and CDK1, CDK1 was chosen as target gene in further experiment. The results of qRT-PCR showed that the expression level of CDK1 in SK-HEP-1, Huh-7, and BEL-7404 cell lines was significantly higher than in MHCC97-L cell line (*P*<0.05) (Supplementary Figure 1A), which indicated that HEP3B2.1-7 and SK-HEP-1 cell lines were more appropriate to research the functions of CDK1. The results of qRT-PCR showed that the knockdown efficiency of CDK1 in shCDK1-1 group is 99.7% compared with that in shCtrl group (*P*<0.01) (Supplementary Figure 1B). Therefore, CDK1-1 was chosen as effective interference target. In order to further verify the functions of DEPDC1B in HCC lines, investigate the functions of CDK1 knockdown in HCC lines, and find the interaction between CDK1 and DEPDC1B, LV-NC(OE+KD), LV-DEPDC1B+NC(KD), LV-shCDK1+NC(OE), and LV-DEPDC1B+shCDK1 were transfected into SK-HEP-1 cell line. The fluorescence of cells, which were infected with LV- NC(OE+KD), DEPDC1B+NC(KD), shCDK1+NC(OE), and DEPDC1B+shCDK1 for 72 h, observed by microscope demonstrates a >80% efficiency of infection (Supplementary Figure 1C). According to the instructions, the efficiency and expression level were tested by qRT-PCR and Western blot, and the results indicated that these cell lines were constructed successfully (Supplementary Figure 1D, 1E).

### DEPDC1B regulates HCC progression mediated by CDK1

**Figure 6:**
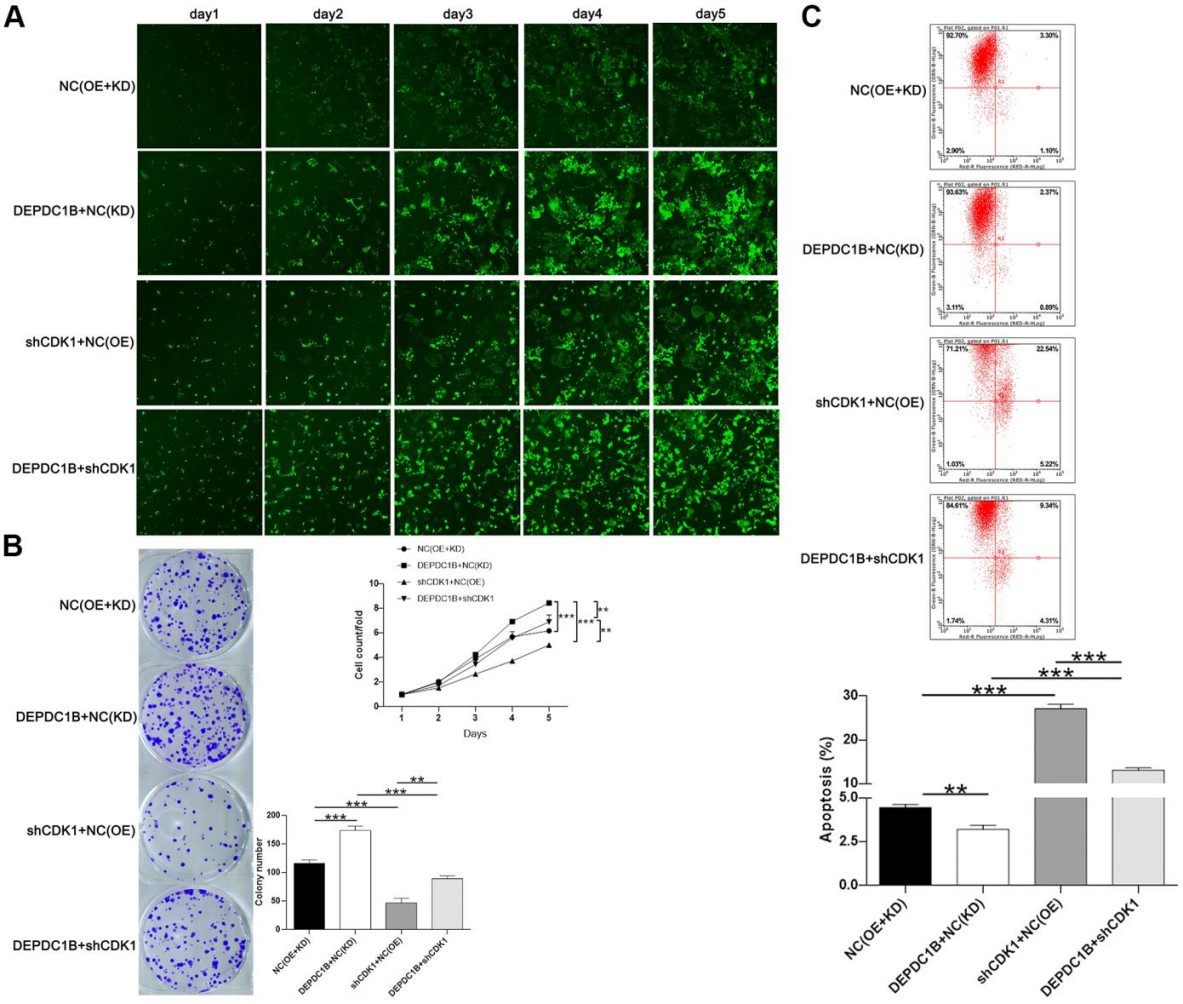
**Functions on cell progression.** (**A**) The results of Celigo cell counting assay show that, compared with NC(OE+KD) group: the cells in DEPDC1B+NC (KD) group exhibited faster proliferation rate (P<0.001), and the cells in shCDK1+NC(OE) group exhibited slower proliferation rate (P<0.001). Compared to DEPDC1B+NC(KD) group, the cells in DEPDC1B+shCDK1 group exhibited slower proliferation rate (P<0.01). The cells in DEPDC1B+shCDK1 group exhibit faster proliferation rate, compared with shCDK1+NC(OE) group (P<0.01). (**B**) The results of colony formation assay show that, compared to NC(OE+KD) group: the cell colony number in DEPDC1B+NC(KD) group was significantly increased (P<0.001), while the cell colony number in shCDK1+NC(OE) group was significantly decreased (P<0.001). Compared with DEPDC1B+NC(KD) group, cell colony number was significantly decreased in DEPDC1B+shCDK1 group (P<0.001). Cell colony number in DEPDC1B+shCDK1 group was significantly increased, compared to shCDK1+NC(OE) group (P<0.01). (**C**) The results of flow cytometry demonstrate that: compared to NC(OE+KD) group: cell apoptosis was decreased in DEPDC1B+NC(KD) group (P<0.01), and in shCDK1+NC(OE) group, cell apoptosis was significantly increased (P<0.001). Compared with DEPDC1B+NC(KD) group, cell apoptosis was significantly increased in DEPDC1B+shCDK1 group (P<0.001). Compared to shCDK1+NC(OE) group, cell apoptosis was decreased in DEPDC1B+shCDK1 group (P<0.001). **: P <0.01. ***: P <0.001.

**Figure 7:**
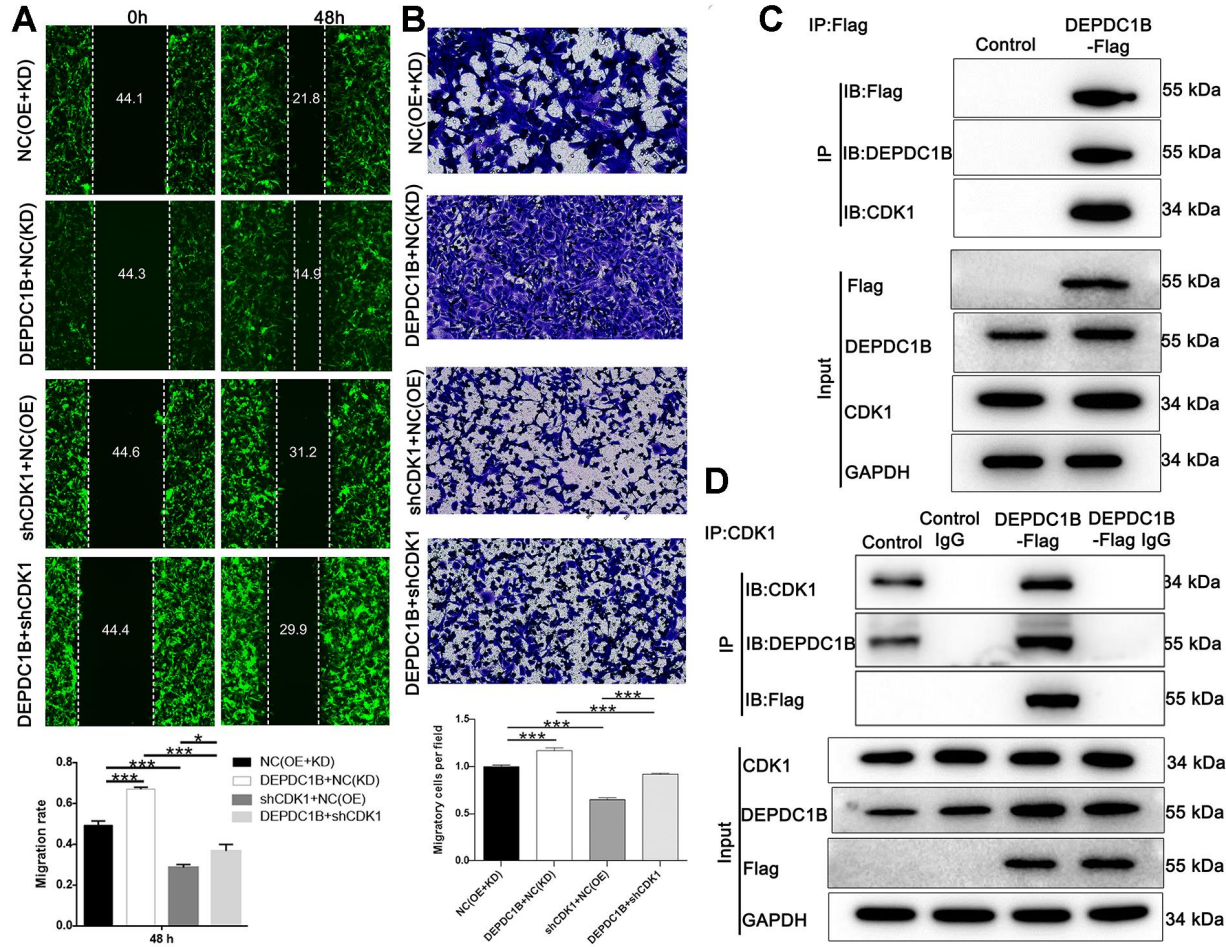
**Detection of cell migration ability, and interaction between CDK1 and DEPDC1B.** (**A**) The results of wound-healing assay and Transwell assay showed that, compared to NC(OE+KD) group: the migration rate of cells was significantly increased in DEPDC1B+NC(KD) group (48 h) (P<0.001), while in shCDK1+NC(OE) group (48 h), the migration rate of cells was significantly decreased (P<0.001). Compared to DEPDC1B+NC(KD) group, the migration rate of cells was significantly decreased in DEPDC1B+shCDK1 group (48 h) (P<0.001). Compared with shCDK1+NC(OE) group, the migration rate of cells was significantly increased in DEPDC1B+shCDK1 group (48 h) (P<0.05). (**B**) The transwell assay shows that, compared to NC(OE+KD) group: the migration ability of cells in DEPDC1B+NC(KD) group was significantly increased (P<0.001), and the migration ability of cells in shCDK1+NC(OE) group is significantly decreased (P<0.001). Compared to DEPDC1B+NC(KD) group, the migration ability of cells was significantly decreased in DEPDC1B+shCDK1 group (P<0.001). Compared with shCDK1+NC(OE) group, the migration ability of cells in DEPDC1B+shCDK1 group was significantly increased (P<0.001). (**C**, **D**) The results of co-immunoprecipitation. *: P <0.05. **: P <0.01. ***: P <0.001.

The results showed that CDK1 knockdown inhibited cell proliferation, while DEPDC1B overexpression promoted cell proliferation (Figure 6A). In terms of colony formation, CDK1 knockdown significantly inhibited cell colony formation, and to the contrast, DEPDC1B overexpression significantly promoted cell colony formation (Figure 6B). CDK1 knockdown promoted cell apoptosis, and DEPDC1B overexpression inhibited cell apoptosis (Figure 6C). The migration ability of cells was significantly increased due to DEPDC1B overexpression, on the contrary, CDK1 knockdown significantly inhibited the migration ability (Figure 7A, 7B). The results of co-immunoprecipitation were displayed in Figure 7C, 7D and showed that DEPDC1B can interact with CDK1. According to the results above, CDK1 knockdown inhibited cell proliferation, colony formation, migration, and promoted cell apoptosis, which indicated CDK1 knockdown suppressed the progression of HCC cell lines. As shown in the results above, DEPDC1B knockdown inhibited the progression of HCC cell lines. When DEPDC1B was overexpressed, the progression in HCC lines was promoted, which suggested that DEPDC1B certainly played a part in HCC progression. At the same time, in DEPDC1B+shCDK1 group, the results showed that the progression was inhibited in HCC cell lines, which suggested the promoting functions of DEPDC1B overexpression on HCC progression may be blocked after CDK1 knockdown. These results showed that CDK1 may be downstream gene of DEPDC1B, and the function of DEPDC1B on HCC progression was regulated by the expression of CDK1. Collectively, the results proved that DEPDC1B played a definite role in the HCC progression, and CDK1 played a key role in DEPDC1B regulating HCC progression. Based on the TCGA database, the correlation between CDK1 and DEPDC1B was analyzed with Pearson Correlation (R=0.72, *P*<0.001). The expression level of CDK1 was correlated with pathologic T, N, M, tumor stage, and gender in the analysis of TCGA on lung adenocarcinoma (Table 5).

**Table 5:** The relationship between the expression of CDK1 and tumor characteristics in patients with lung adenocarcinoma under TCGA.

		**CDK1 expression**		**No.**	***P***
		**Low**	**High**		
Pathologic_T	T1	100	67	167	0.016
	T2	114	145	259	
	T3	22	23	45	
	T4	9	10	19	
Pathologic_N	N0	173	145	318	0.010
	N1	37	54	91	
	N2+N3	28	44	72	
Pathologic_M	M0	154	170	324	0.007
	M1	5	20	25	
	M2	0	0	0	
Stage	Stage_I	148	115	263	0.003
	Stage_II	53	63	116	
	Stage_III	35	45	80	
	Stage_IV	6	20	26	
Gender	female	149	117	266	0.004
	male	98	129	227	
Age	low	117	133	250	0.137
	high	130	113	243	

### Effects of DEPDC1B knockdown on tumor growth *in vivo*

**Figure 8:**
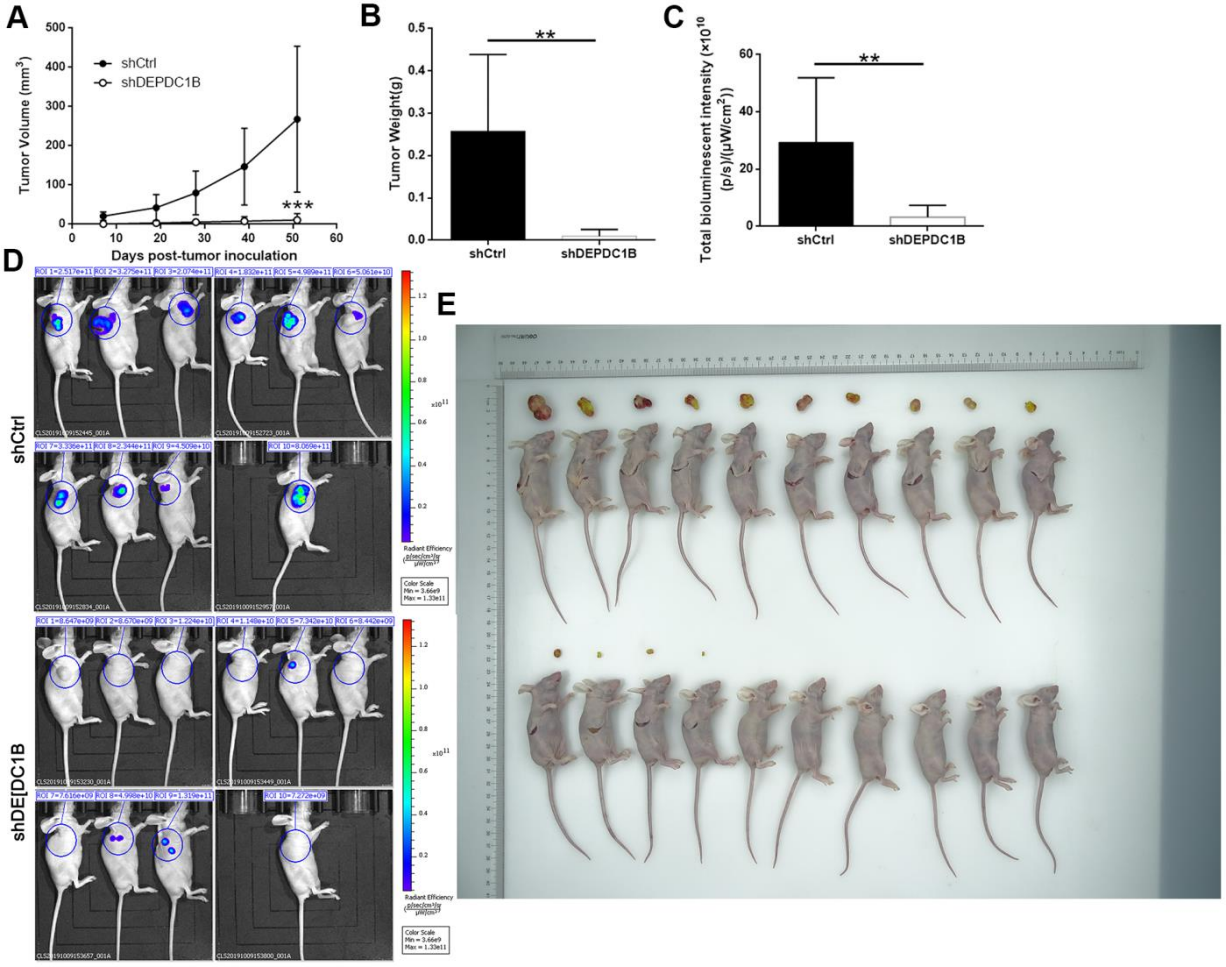
**Influences of DEPDC1B knockdown on tumor *in vitro*.** The volumes of tumors were obviously decreased in shDEPDC1B group (**A**). The weight (**B**) and fluorescence intensity (**C**) of tumors in shDEPDC1B were significantly lighter and weaker than that in shCtrl group (P<0.01). (**D**, **E**) Images of mice were taken under small animal living imaging system and digital camera. **: P <0.01.

The mice xenograft model was built with BALB/c nude mice to detect the effects of DEPDC1B knockdown on the tumorigenicity of SK-HEP-1 cells. After 51 days, the final weight and volume of tumors in mice was measured after the tumors were resected. The results showed that the volumes of tumors in shHEP3B2.1-7 group were smaller than that in shCtrl group (Figure 8A), and the weight of tumors in shHEP3B2.1-7 group were significantly lighter than that in shCtrl group (*P*<0.01) (Figure 8B). The data about fluorescence intensity was measured under small animal living imaging system. The bioluminescence intensity in shHEP3B2.1-7 group was significantly weaker, compared to shCtrl group (*P*<0. 01), which suggested the slower growth of tumors after DEPDC1B knockdown. (Figure 8C–8E). Collectively, the results above showed the slower growth of HCC cells in shDEPDC1B group compared with that in shCtrl group.

## DISCUSSION

In 2012, 782500 new liver cancer cases and 745500 deaths occurred in the world and 50% of the total cases happened in China. Presently, five means are used in HCC to achieve the best outcome (surgical resection, transplantation, local ablation, transarterial embolization and radiotherapy (TACE), and systemic pharmacological treatment), in which, surgical resection, transplantation, and local ablation are treated as potentially curative treatment for only early-stage HCC patients and widely used in the world [4, 7, 13]. TACE can be chosen as an effective treatment option in intermediate-stage HCC patients [14]. In systemic pharmacological treatment, sorafenib (inhibitor of VEGFR1, VEGFR2 and VEGFR3) is the first-line systemic treatment of patients with advanced-stage HCC, and results of phase III randomized controlled trials have proved that the function of sorafenib in prolonging the survival of advanced-stage HCC patients [4, 13]. More and more researchers pay more attention to the occurrence, progression, and recurrence, strive to find specific genes and mechanism to cure HCC. More and more chemotherapy and molecular targeted drugs were found effective in advanced-stage HCC patients: regorafenib (similar to sorafenib) [15, 16]; lenvatinib (inhibitor of VEGFR1–3, FGFR1–4, PDGFRα, RET, and KIT) [4]; cabozantinib (inhibitor of MET, VEGFR2, AXL, RET, and KIT) [17]; ramucirumab (monoclonal antibody targets against VEGFR2) [18]; and nivolumab (inhibitor of programmed cell death protein-1 immune checkpoint) [19]. However, the recurrence and mortality rate remained high. Therefore, further research about the molecular mechanism is important for finding the effective treatment of HCC.

The functions of DEPDC1B were investigated in this study. DEPDC1B is localized to the adhesion sites between cells and contains two structural domains: a DEP domain and a RhoGAP domain [11, 20]. DEPDC1B can be treated as a negative regulator in RhoA/ROCK signaling pathway, due to the decreased expression of RhoA and MLC2 (main target of ROCK) in DEPDC1B knockdown cells [21]. After DEPDC1B silencing, the delay in mitosis was occurred, especially at the point from G2 to M, and probably caused by defects in de-adhesion. The delay could be rescued by RhoA silencing or inhibiting ROCK [21]. DEP domain is often found in proteins which participate in signal transduction and interact with various membrane partners (phospholipids and membrane receptors), which is necessary for Wnt signaling [22, 23]. It plays important roles in mediating membrane localization, and regulating wide range cell functions [24, 25]. RhoGAP domain can interact with GTPase and small GTPase, responsible for Rho GTPase signaling [26]. Yang Y, et al [27] showed that DEPDC1B can tumor cell migration and invasion through activating Wnt/β-catenin signaling pathway in non-small cell lung cancer. Ahuja P and Singh K [28] showed that DEPDC1B promoted oral cancer cell growth, invasion, and anchorage independent growth mediated by the interaction between DEPDC1B and Rac1. However, the functions of DEPDC1B in HCC has never been investigated. The study is the first research displaying the functions and mechanism of DEPDC1B in HCC. The results of experiments showed that DEPDC1B knockdown of DEPDC1B inhibited cell proliferation and migration, which is consistent with other reports involving functions of DEPDC1B in other cancers [10]. Collectively, DEPDC1B can regulate the HCC cells growth *in vitro* and *vivo* and it can be regarded as new genetic target to instruct HCC therapy.

In IPA analysis, DEPDC1B can affect various genes in Cyclins and Cell Cycle Regulation and Wnt/β-catenin Signaling pathway through EED and FANCD2. In above genes, the expression of CCNE2, CDK1, CDK6, and E2F1 were down-regulated after DEPDC1B knockdown. The results of gene recovery experiments showed that promotion effect of DEPDC1B on cell proliferation and migration in HCC cells could be rescued by CDK1 knockdown. The results of Co-IP indicated the interaction between DEPDC1B protein and CDK1 protein in SK-HEP-1 cells. The results of human apoptosis antibody array showed that the expression of Caspase3, p21, and IGFBP-6 was up-regulated, and Bcl-2, Bcl-w, HSP27, HSP70, Livin, sTNF-R2, TNF-α, and TNF-β was down-regulated. CDK1 is a member of the Ser/Thr protein kinase family and known as M-phase promoting factor (MPF), which plays important roles in eukaryotic cell G1/S and G2/M phase transitions. It was thought that the activation of CDK1 by A-type cyclins at the end of interphase promoted mitotic entry [29]. CDK1 can cooperate with cyclins A and B to promote S, G2, and M phase progression, CHK1 and WEE1 can inhibit CDK1 through inhibitory phosphorylation and CDC25 family [12, 30, 31], therefore the down-regulated CDK1 and CCNB coordinated to lead G2/M phase in the study. In summary, these results in the study proved that DEPDC1B can regulated HCC cell progression based on the CDK1. If CDK1 was knockdown, the pathway of DEPDC1B would be inhibited or blocked, which was consistent the results in other cancers. Caspase3 is a member of the caspase family, which was thought essential regulators in programmed cell apoptosis [32, 33]. The cells in hepatoma and various phosphorylated forms can secret IGFBP-6. IGFBP-6 has stronger affinity for IGF-II than IGF-II, and the inhibiting cell function was thought to achieve through IGF binding [34]. p21 is an inhibitor of CKI and inhibitor of all cyclin/CDK complexes. p21 can inhibit G1/S at the mitotic entry, and it can be activated by p53 to link DNA damage to cell cycle arrest [35]. Study has shown that p21 was related to cell differentiation, tumor growth, and metastatic potential in melanoma [36]. Bcl-2 and Bcl-w are members of BCL-2 family, which plays an important role in programmed cell apoptosis as inhibitor of cell apoptosis and cell cycle entry, and regulator of anti-apoptotic intracellular signals [37, 38]. HSP27 is a partner of sHSP, and it is highly expressed in many cancers, which displays the functions of promoting cell migration, invasion, and inhibiting cell apoptosis [39, 40]. At the same time, Hsp70 has the functions of binding to unfolded protein, trafficking protein, and regulating enzyme activity, functions as regulators of tumor proliferation, migration, cell apoptosis [40–42]. Livin (known as BIC7) is a member of IAP family, which plays an important role in the sensitivity of chemotherapy to cancers, and the expression level was related to the tumor progression [43]. TNF-α and TNF-β are members of TNF family, the functions of TNF consist of cell proliferation, differentiation, and apoptosis. Collectively, DEPDC1B knockdown induces cell apoptosis, leads G2 phase arrest, and inhibit growth and migration in tumor mediated by CDK1.

## CONCLUSIONS

In conclusion, this is the first study concerning about DEPDC1B in HCC development. The study confirmed that DEPDC1B is overexpressed in hepatic carcinoma tissues, and the development of HCC were regulated by DEPDC1B through controlling cell apoptosis, inducing G2 phase arrest, migration, and proliferation. It can interact with CDK1, and played a key role in DEPDC1B regulating HCC progression. The study has proved that DEPDC1B play an important role in the progression of HCC and can be treated as a novel gene target to instruct the treatment of HCC. Thus, further studies may accelerate our understanding about the functional role of DEPDC1B in HCC.

## MATERIALS AND METHODS

### Patients and tissue samples

A total of 178 cases (collect 2 slides from each tissue and weed out 2 off slides) diagnosed as HCC by preoperative image examinations and postoperative immunohistochemistry in The First Affiliated Hospital of Zhengzhou University were gathered in the study. This study had got approval from Ethics committees of the First Affiliated Hospital of Zhengzhou University and written informed consent was acquired from all participants in the study.

### Immunohistochemistry

The immunohistochemistry assay was conducted to detect the expression level of DEPDC1B and CDK1 in hepatocellular carcinoma tissues and para-carcinoma tissues according to the manufacturer’s instructions. The slides were made from tissues according to the routine slides preparation procedure and then incubated with primary antibody (1:200, Cat. No. bs-14278R, BIOSS, Beijing, China) against DEPDC1B at 4° C overnight. The secondary antibody was added to interact with the anti-DEPDC1B antibody at 37° C for 1 h. The 3, 3’-diaminobenzidine (DAB) was used as developer and then the expression levels of DEPDC1B were detected with the assistance of inverted microscope (Olympus IX73). The positive cells score was classified on the basis of the proportion of positive cells: 0 (0%), 1 (10-25%), 2 (25-50%), 3 (50-75%), and 4 (75-100%). Staining intensity score was graded as 0 (No color), 1 (Buff), 2 (Brownish yellow), and 3 (Dark brown) according to the color of cytoplasm, membrane, nucleus, and stroma. And the score of expression levels were: 0, negative; 1-4, positive; 5-8, strong positive; and 9-12, stronger positive, based on the sum of positive cells score and staining intensity score.

### Construction of lentivirus for shRNA

Multiple RNAi targeting sequences were designed based on template gene DEPDC1B (NM_018369) and CDK1 (NM_001170407) according to the design principles of RNAi sequences. After evaluation by the BLAST program for homology search, the sequence DEPDC1B (5’-GCTGCTAGATTGGTAACGTTT-3’), Human-CDK1-1 (5’-TTCCATGGATCTGAAGAAATA-3’), Human-CDK1-2 (5’-AGACTAGAAAGTGAAGAGGAA-3’), and Human-CDK1-3 (5’-ATGGAGTTGTGTATAAGGGTA-3’) were chosen as specific interference target. The shRNA sequences were designed based on the selected specific sequence and for the purpose of subsequent vector construction, appropriate restriction enzyme sites were added to the shRNA sequences. Eventually, the sequences were completed and listed in Supplementary Table 1. Thereafter, the dsDNA oligo was prepared for connecting the plasmid BR-V-108 and LV-007 linearized by AgeI (Cat. No. R3552L, NEB, Beijing, China), EcoR (Cat. No. R3552L, NEB, Beijing, China), and BamHI according to the manuals. Finally, the positive clones were sequenced and confirmed the veracity of shRNA’s sequence, plasmids were abstracted from the competent cells and purified to package the lentiviruses (LV). The recombinant lentiviruses expressing DEPDC1B shRNA, CDK shRNA, over-expressing DEPDC1B, scrambled shRNA, and vector LV-007 were named as LV-shDEPDC1B, LV-shCDK1, LV-DEPDC1B, LV-shCtrl, and LV-Control, respectively.

### Establishment of cell lines and Celigo cell counting assay

Cell lines which expressed more DEPDC1B and CDK1 were selected among HEP3B2.1-7, SK-HEP-1, huh-7, MHCC97-L, BEL-7404, and HCCLM3 cell lines by qRT-PCR for subsequent study. The newly produced LV-shDEPDC1B and LV-shCtrl were used to infected the cell lines SK-HEP-1 and HEP3B2.1-7. SK-HEP-1 cell line was infected by LV-NC (OE+KD), LV-DEPDC1B+NC(KD), LV-shCDK1+NC(OE), and LV-DEPDC1B+shCDK1.. Cells in logarithmic growth phase were digested by 0.05% Trypsin-EDTA to make into cell suspension and cultured in the medium consisting of RPMI 1640 and 10% FBS for 24 h. When the cells achieved the level of 2×10^5/well, the lentivirus (40 μL, 1×10^8 TU/mL) were used to infected the cells in ENI. S+Polybrene (MOI 20) for 20 h. Finally, the expression of GFP (green fluorescent protein) was observed with the assistance of fluorescence microscope for detecting the efficiency of cell infection. When the cell fusion in NC (OE+KD) group, DEPDC1B+NC(KD) group, shCDK1+NC(OE) group, and DEPDC1B+shCDK1 group reached 80%, the Celigo cell counting assay was used to detect the influence of DEPDC1B and CDK1 on cell proliferation according to the manufacturer’s instructions. The number of cells were detected with Celigo and analyzed to obtain Fold Change, finally.

### Quantitative RT-PCR

Total RNA was collected from all group cells after being lysed by Trizol (Cat. No. 15596-026, Invitrogen, Carlsbad, California, USA) based on the manuals. GAPDH was chosen as the reference gene in the study, the primer sequence of GAPDH, DEPDC1B, CDK1 were listed in Supplementary Table 2. The cDNA was obtained by RNA reverse transcription with M-MLV kit purchased from Promega, respectively. Then the real-time qPCR was conducted in two-step method to obtain the Ct value of GAPDH, DEPDC1B, and CDK1 according to the instructions. Average ΔCt was used to detect the DEPDC1B and CDK1 expression abundances in SK-HEP-1 and HEP3B2.1-7 cell lines and relative quantitative analysis was used to detect the expression level of DEPDC1B and CDK1.

### Western blot and co-immunoprecipitation

The density of total protein was measured with BCA Protein Assay Kit (Cat. No. 23225, HyClone-Pierce, Guangzhou, China) to meet the standard of 20ug protein/well in subsequent loading and perform the experiment according the instructions. The blocked membrane was incubated with primary antibodies (Supplementary Table 3) for 2 h at room temperature. The secondary antibody (Supplementary Table 3) was added to interact with PVDF membrane for 2 h at room temperature after membrane being washed with TBST for 3 times. ECL+ plusTM Western blotting system kit was used to stain the membrane for the following X-ray developing.

### MTT assay

Cells in logarithmic growth phase were digested by pancreatic enzymes and suspended with the complete DMEM then allocated to 96-well plates (2000 cell/well) for 3 replicates. From the next day, cells were incubated with MTT for 4 h before detecting the OD value. The OD value (490/570 nm) was detected under microplate reader (Cat. No. M2009PR, Tecan infinite, Mannedorf, Switzerland) in 5 days according to the instructions.

### Colony formation assay

The cells in NC (OE+KD) group, DEPDC1B+NC(KD) group, shCDK1+NC(OE) group, and DEPDC1B+shCDK1 group were trypsinized and resuspended in logarithmic growth phase, and cells number was counted. 500 cells/well in each group were reseeded into six-well plates in triplicate. Cells were incubated for 8 days, and the medium was displaced every 3 days. 1 mL paraformaldehyde was added into well for 50 min to fix cells. 500 μL GIEMSA staining was added into each well for 20 min after cells were washed with PBS. Finally, the photos of colony cells were taken by microscope equipped with digital camera.

### Flow cytometry assay

The cell apoptosis was induced by apoptosis kit (Cat. No. 88-8007-74, eBioscien, California, USA) when cells are grown to coverage of 70% in 6-well plate. The cell in well was digested by pancreatin and suspended by complete medium. After washed with D-Hanks (pH=7. 2~7. 4), the cell sedimentation should be resuspended with 200 μl 1×binding buffer. The solution containing 200 μL suspension and 10 μL annexin V-APC was kept away from light for 15 min. Finally, the cells were analyzed by flow cytometry system (Cat. No. Guava easyCyte HT, Millipore, MIT, USA).

### Cell cycle analysis

The analysis of cell cycle was detected under flow cytometry system (FACSCalibur, Becton, Dickinson and Company, New Jersey, USA). The cells were washed with D-Hanks after discarding the supernatants, when the cell coverage was up to 80% of 6 cm dish. The cells were gathered at 1500 rpm for 5 min after washed by 4° C PBS. The gathered cells were fixed by 4° C 70% ethanol for 1 h. Then the fixed cells were resuspended with 1.5ml staining solution consisting of 40×PI, 1×PBS, and 100×RNase formulated at a ratio of 25: 10: 1000. The passes rate of resuspended cells was controlled to 200-350 cells/s under Flow cytometry system.

### Wounding healing assay

The wounding healing assay was used to test the migration ability of transfected cells according to the instructions. SK-HEP-1 cells and HEP3B2.1-7 cells were cultured in 96-well plate. The concentration of serum in medium was lowered when the confluence of cells reached 90% on the second day, and according to the instructions of 96 wounding replicator (Cat. No. VP408FH, VP scientific, Shanghai, China) to form wounds. The migration distance and rate of SK-HEP-1 cells and HEP3B2.1-7 cells were detected under fluorescence microscope after incubated with DMEM+0.5% PBS for 8 h, 24 h, 32 h and 48 h.

### Transwell assay

The 5×10^4/well SK-HEP-1 cells and 10×10^4/well HEP3B2.1-7 cells in period of logarithmic growth were digested by trypsin and resuspended in low concentration serum medium. The resuspension was diluted by serum-free medium and 100 μL diluent was seeded into 24-well plate (upper chamber). The upper chamber was placed in the lower chamber with 600 μL medium contained 30% FBS. SK-HEP-1 cells and HEP3B2.1-7 cells were cultured for 16 h, 24 h, and 48 h, respectively. 400 μL staining solution was added to interact with the cells for 20 min after SK-HEP-1 cells and HEP3B2.1-7 cells were cultured for 16 h, 24 h and 48 h, respectively and the invading cells were counted under fluorescence microscope.

### Human apoptosis antibody array

SK-HEP-1 cells were chosen to study the general mechanism of DEPDC1Bknockdown under human apoptosis antibody array (Cat. No. ab134001, Abcam, Cambridge, MA, USA) according to the manufacturer’s instructions. The expression level of genes in human apoptosis pathway was examined to obtain significant results.

### Human GeneChip PrimeView

The total RNA of was extracted from SK-HEP-1-shCtrl group and SK-HEP-1-shDEPDC1B group in three times for subsequent study. Human GeneChip PrimeView was operated to find DEGs (differentially expressed genes) according to the manufacturer’s instructions. The GeneChip was reacted with moderate Pre-Hybridization Mix for 10 min at 45° C. Then, Hybridization Master Mix containing fragmented and labeled cRNA was reacted with the GeneChip for 16 h at 45° C 60rpm. Finally, the GeneChip was scanned under GeneChip Scanner, and analyzed by matched GeneChip software. Quality evaluation of chip data was measured by signal histogram, relative log expression plot, correlation analysis, and principal component analysis. The significant P-value was calculated by linear model based on empirical Bayesian distribution and corrected to obtain FDR. |Fold Change|≥ 1.5 and FDR< 0.05 between two groups were regarded as criteria to screen DEGs. DEGs were analyzed with volcano plot and hierarchical clustering plot. Ingenuity Pathway Analysis (IPA) was used in classic pathways analysis, upstream regulation analysis, disease and function analysis, and interaction network analysis among the DEGs.

### Mice xenograft model

The mice xenograft model was created with 4-weeks nude mice (BALB/c, female, Charles River, Beijing, China) according to the manual instructions and the experimental procedure continued for 51 days. A total of 20 mice were randomly divided into two groups before mice were injected cells into their front legs. 4×10^6 SK-HEP-1 cells/mouse transfected with LV-shDEPDC1B and LV-shCtrl were injected into the mice in shDEPDC1B group and shCtrl group, respectively. The length, width, and fluorescence intensity of tumors were detected with small animal living imaging system after mice had been anesthetized with intraperitoneal injection of 0.7% sodium pentobarbital. At the final of experiment, tumors in mice were resected to detect the weight and calculate volume (based on the formula: volume = length × width^2 × 3.14/6) after being sacrificed with excessive sodium pentobarbital followed by cervical dislocation method. All the animal experimental protocols were approved by the Ethics Committee at the First Affiliated Hospital of Zhengzhou University.

### Statistical analysis

Statistical analysis of data was performed with SPSS 22.0 software. These experimental procedures were operated in triple. Student’s t-test, Mann-Whitney U test, and Pearson Correlation were performed to analyze continuous and categorical variables from the experiment. All values in the text and figures were expressed as the mean ± standard deviation. All statistical tests were two-tailed, and *P* value <0. 05 was considered statistically significant.

### Availability of data and materials

The main datasets used and analyzed in the present study are available from the corresponding authors on reasonable request.

### Ethics approval and consent to participate

Written informed consent was gained from all patients whose tissue samples were used in this study and this study was approved by Ethics committees of the First Affiliated Hospital of Zhengzhou University. All the animal experimental protocols were approved by the Ethics committee at the First Affiliated Hospital of Zhengzhou University.

## Supplementary Material

Supplementary Figure 1

Supplementary Tables
