# Supplementary material for: Overexpressed DEPDC1B contributes to the progression of hepatocellular carcinoma by CDK1
Source: Aging (Albany NY). 2021 May 25;13(16):20094–115. doi: 10.18632/aging.203016 (PMC8436915; doi:10.18632/aging.203016)
Supplement: Supplementary Figure 1 [file aging-13-203016-s001.pdf]

SUPPLEMENTARY FIGURE

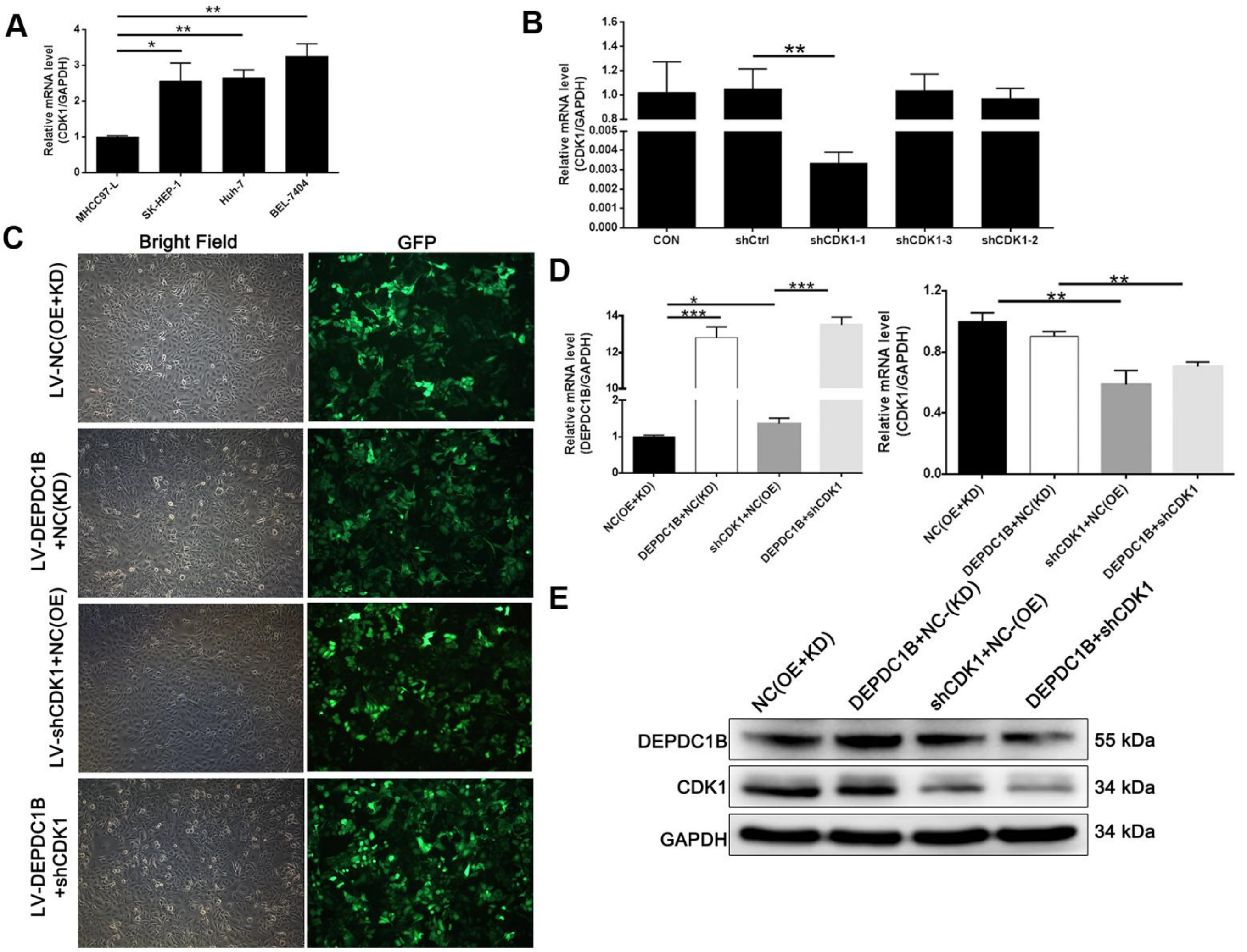

Supplementary Figure 1. Successful establishment of CDK1 knockdown and DEPDC1B overexpression. (**A**) Compared with the MHCC97-L cells, CDK1 is highly expressed in SK-HEP-1, Huh-7 and BEL-7404 cells (P<0.05). (**B**) The results of qRT-PCR show that, in SK-HEP-1 cells, after the infection of lentivirus, compared with the shCtrl group: The knockdown efficiency of CDK1 in shCDK1-1 group is 99.7% (P<0.01). (**C**) The fluorescence of cells, which were infected with 4 groups for 72 h, observed by microscope demonstrates a >80% efficiency of infection and the normal cell condition. (**D**) After infection, compared to NC group:DEPDC1B mRNA level of DEPDC1B+NC(KD) group increased obviously (P<0.001), but CDK1 mRNA level had no significant change, and DEPDC1B mRNA level of shCDK1+NC(OE) group increased obviously (P<0.05), but CDK1 mRNA level decreased obviously (P<0.01). Compared to DEPDC1B+NC(KD) group, DEPDC1B mRNA level of DEPDC1B+shCDK1 group had no significant change, but CDK1 mRNA level decreased obviously (P<0.01). Compared with shCDK1+NC(OE) group, DEPDC1B mRNA level of DEPDC1B+shCDK1 group increased obviously (P<0.001), but CDK1 mRNA level had no significant change. (**E**) The results of Western blot show that, compared to NC group: DEPDC1B protein level of DEPDC1B+NC(KD) group increased obviously, CDK1 protein level had no significant change, and DEPDC1B protein level of shCDK1+NC(OE) group increased obviously, but CDK1 protein level decreased obviously. Compared with DEPDC1B+NC(KD) group, DEPDC1B and CDK1 protein level of DEPDC1B+shCDK1 group decreased obviously. Compared to shCDK1+NC(OE) group, DEPDC1B and CDK1 protein level of DEPDC1B+shCDK1 group decreased obviously. *: P <0.05. **: P <0.01. ***: P <0.001.
