# Supplementary material for: Overexpressed DEPDC1B contributes to the progression of hepatocellular carcinoma by CDK1
Source: Aging (Albany NY). 2021 May 25;13(16):20094–115. doi: 10.18632/aging.203016 (PMC8436915; doi:10.18632/aging.203016)
Supplement: Supplementary Tables [file aging-13-203016-s002.pdf]

SUPPLEMENTARY TABLES

Supplementary Table 1. shRNA sequences for vector construction.

Target gene	Code	Primer (5'-3')
DEPDC1B knock down		F: CCGGGCTGCTAGATTGGTAACGTTTCTCGA GAAACGTTACCAATCTAGCAGCTTTTTG<br>R: AATTCAAAAAGCTGCTAGATTGGTAACGTT TCTCGAGAAACGTTACCAATCTAGCAGC
CDK1 knockdown	1	F: CCGGTTCCATGGATCTGAAGAAATACTCGA GTATTTCTTCAGATCCATGGAATTTTTG<br>R: AATTCAAAAATTCCATGGATCTGAAGAAAT ACTCGAGTATTTCTTCAGATCCATGGAA
	2	F: CCGGAGACTAGAAAGTGAAGAGGAACTCGA GTTCCTCTTCACTTTCTAGTCTTTTTTG<br>R: AATTCAAAAAAGACTAGAAAGTGAAGAGGA ACTCGAGTTCCTCTTCACTTTCTAGTCT
	3	F: CCGGATGGAGTTGTGTATAAGGGTACTCGAG TACCCTTATACACAACTCCATTTTTTG<br>R: AATTCAAAAAATGGAGTTGTGTATAAGGGTAC TCGAGTACCCTTATACACAACTCCAT
DEPDC1B overexpression		F: GATTCTAGAGCTAGCGAATTCCGCCACCATGG AGCATCGCATCGTGGGGCC<br>R: TCCTTCGCGGCCGCGGATCCTTACATTCGAAAA CTTCTAGTTCTTTGAAATGG

F, Forward; R, Reverse.

Supplementary Table 2. Primer sequences for quantitative PCR.

Target gene	Primer (5'-3')
GAPDH	F: TGACTTCAACAGCGACACCCA<br>R: CACCCTGTTGCTGTAGCCAAA
DEPDC1B	F: CTGAAGTGACCCGCAAACAAA<br>R: CTGGTGGGAGATCATTCCATTC
CDK1	F: CCATACCCATTGACTAACTAT<br>R: ACCCCTTCCTCTTCACTTTC

F, Forward; R, Reverse.

Supplementary Table 3. Characteristics of antibodies used in the study.

Antigen	Application	Origin	Cat. No.	Dilution
Primary antibodies				
DEPDC1B	WB, Co-IP	Abcam, Cambridge, MA, USA	ab124182	1:1000
GAPDH	WB, Co-IP	Bioworld	AP0063	1:3000
Akt	WB	CST, Danvers, MA, USA	4685	1:1000
P-Akt	WB	BIOSS, Beijing, China	bs-5193r	1:1000
Cyclin D1	WB	CST, Danvers, MA, USA	2978	1:2000
PIK3CA	WB	Abcam, Cambridge, MA, USA	ab40776	1:1000
CDK6	WB	Abcam, Cambridge, MA, USA	ab151247	1:1000
CCNE2 (Cyclin E2)	WB	Abcam, Cambridge, MA, USA	ab40890	1:2000
CDK1	WB, Co-IP	Abcam, Cambridge, MA, USA	ab133327	1:1000
E2F1	WB	Abcam, Cambridge, MA, USA	ab179445	1: 2000
DYKDDDDK Tag	CO-IP	CST, Danvers, MA, USA	14793	1:50
Secondary antibodies				
Goat Anti-Rabbit	WB, Co-IP	Beyotime, Shanghai, China	A0208	1:3000

WB, Western blotting; Co-IP, co-immunoprecipitation.
